# Genomic Engineering in Human Hematopoietic Stem Cells: Hype or Hope?

**DOI:** 10.3389/fgeed.2020.615619

**Published:** 2021-01-22

**Authors:** Stefanie Klaver-Flores, Hidde A. Zittersteijn, Kirsten Canté-Barrett, Arjan Lankester, Rob C. Hoeben, Manuel A. F. V. Gonçalves, Karin Pike-Overzet, Frank J. T. Staal

**Affiliations:** ^1^Department of Immunology, Leiden University Medical Center, Leiden, Netherlands; ^2^Department of Cell and Chemical Biology, Leiden University Medical Center, Leiden, Netherlands; ^3^Department of Pediatrics, Willem-Alexander Children's Hospital, Leiden University Medical Center, Leiden, Netherlands

**Keywords:** CRISPR-Cas9, gene editing, hematopoietic stem cells, stem cell biology, genomic engineering, therapeutic, clinic

## Abstract

Many gene editing techniques are developed and tested, yet, most of these are optimized for transformed cell lines, which differ from their primary cell counterparts in terms of transfectability, cell death propensity, differentiation capability, and chromatin accessibility to gene editing tools. Researchers are working to overcome the challenges associated with gene editing of primary cells, namely, at the level of improving the gene editing tool components, e.g., the use of modified single guide RNAs, more efficient delivery of Cas9 and RNA in the ribonucleoprotein of these cells. Despite these efforts, the low efficiency of proper gene editing in true primary cells is an obstacle that needs to be overcome in order to generate sufficiently high numbers of corrected cells for therapeutic use. In addition, many of the therapeutic candidate genes for gene editing are expressed in more mature blood cell lineages but not in the hematopoietic stem cells (HSCs), where they are tightly packed in heterochromatin, making them less accessible to gene editing enzymes. Bringing HSCs in proliferation is sometimes seen as a solution to overcome lack of chromatin access, but the induction of proliferation in HSCs often is associated with loss of stemness. The documented occurrences of off-target effects and, importantly, on-target side effects also raise important safety issues. In conclusion, many obstacles still remain to be overcome before gene editing in HSCs for gene correction purposes can be applied clinically. In this review, in a perspective way, we will discuss the challenges of researching and developing a novel genetic engineering therapy for monogenic blood and immune system disorders.

## Introduction

During the last decade, a wide range of scientific advances have emerged in the field of genomic engineering. Those advances vary from γ-retroviruses to self-inactivating lentiviruses, and from designed meganucleases to the more versatile, hence more powerful, CRISPR/Cas-based systems. What makes gene editing technologies interesting for researchers and clinicians, but also for the general public is their potential for therapeutic application in a range of genetic and acquired diseases, such as inborn errors of immunity (IEI) (Gatti et al., 1968), hemoglobinopathies including sickle cell disease (SCD) (Johnson et al., 1984; Lucarelli et al., 1984), cystic fibrosis, certain types of cancers, and viral diseases such as AIDS (White and Khalili, 2016; Shim et al., 2017; Porteus, 2019; Shahryari et al., 2019). However, these promising state-of-the-art technologies face a number of obstacles that prompt questions regarding their safety and efficiency especially when considering clinical applications. Preeminent amongst these obstacles are the generation of off-target effects with associated potential tumorigenicity, and immune responses triggered by the delivery vehicles and/or the gene editing reagents themselves (Doudna and Charpentier, 2014; Shim et al., 2017). In this perspective, we provide a brief overview of hematopoietic stem cell (HSC) biology and *ex vivo* expansion protocols, followed by a critical discussion about the scientific basis for the development of novel HSC gene editing therapies for blood and immune disorders.

## Hematopoietic Stem Cells

Stem cells are cells of embryonic, fetal or adult origin, capable of dividing indefinitely (Staal et al., 2011). All stem cells, regardless of their origin, have three characteristics that distinguish them from other cell types: (i) they are undifferentiated and non-specialized cells; (ii) are able to divide and renew themselves indefinitely; and (iii) are able to differentiate into specialized cells when subjected to certain physiological or experimental conditions. Those cells can be classified, according to their origin or their differentiation capacity, into embryonic and non-embryonic stem cells that can be pluripotent or multipotent, respectively.

Hematopoietic stem cells (HSC) comprise a heterogeneous and relatively small group of cells that have the ability to self-renew and differentiate into specialized cells of the blood tissue and the immune system. Those cells are characterized by being the most immature in the differentiation hierarchy for blood cells.

In the classic model of hematopoiesis, the most primitive HSC progenitor cells (phenotypically defined as CD34^+^ CD38^−^ CD90^+^ CD45RA^−^ and CD49f^−^), differentiate into progenitors that further give rise to other blood cells (Notta et al., 2011). The recently identified Junction adhesion molecule-2 (Jam2) is highly expressed in HSCs and can generate T cells, have been suggested as novel surface markers in HSCs (Radulovic et al., 2019). Also, recently other two molecules have been identified as a relatively robust surface marker in human HSCs. The Endothelial protein C receptor (EPCR) is highly conserved in LT-HSCs (Fares et al., 2017), and the Endothelial cell-selective adhesion molecule (ESAM) is highly expressed in HSCs and MPPs, in a long-term lifetime. Thus, the ESAM seems to have a big influence in HSC differentiation path in different studies (Ooi et al., 2009; Yokota et al., 2009; Ishibashi et al., 2016; Roch et al., 2017).

For clinical applications, the interest of using HSCs has been increasing over the years. Among the difficulties faced by the researchers are the number of cells extracted from the patient, and also the fact that those cells undergo symmetrical and asymmetrical cell divisions when cultured. In an *ex vivo* expansion approach, the symmetrical cell division leads to an increase in the number of cells (Morrison and Kimble, 2006), achieved by the use of different combinations of growth factors and cytokines, such as SCF, TPO, Flt3-L, IL-3, and IL-6 (Sauvageau et al., 2004; Buza-Vidas et al., 2006; Hofmeister et al., 2007; Metcalf, 2008). Aside from that, other compounds are screened and tested for their potential for *in vitro* HSC expansion, including Stemregenin1 (SR1) and UM171 molecules (Boitano et al., 2010; Fares et al., 2014). The SR1 molecule was the first identified with the property of supporting the expansion of human and murine HSCs *in vitro* (Boitano et al., 2010), and has clinical benefit when cultured with the aforementioned cytokines cocktail (Wagner et al., 2016). The UM171 has been shown to be a good and promising candidate for *ex vivo* expansion of human cord blood HSCs (Fares et al., 2014). A recent clinical trial is using the UM171 with the purpose of *ex vivo* expansion of HSCs for allogeneic transplantation and gene therapy (NCT02668315), which suggests the potential use in *ex vivo* gene therapy. An interesting recently identified compound is CPI203, which acts at the epigenetic level to expand human CD34^+^ cells in NSG mouse models and may support *ex vivo* expansion of human HSCs (Hua et al., 2020; Staal and Fibbe, 2020).

## State-of-the-Art Genome Engineering of HSCs

**Figure 1:**
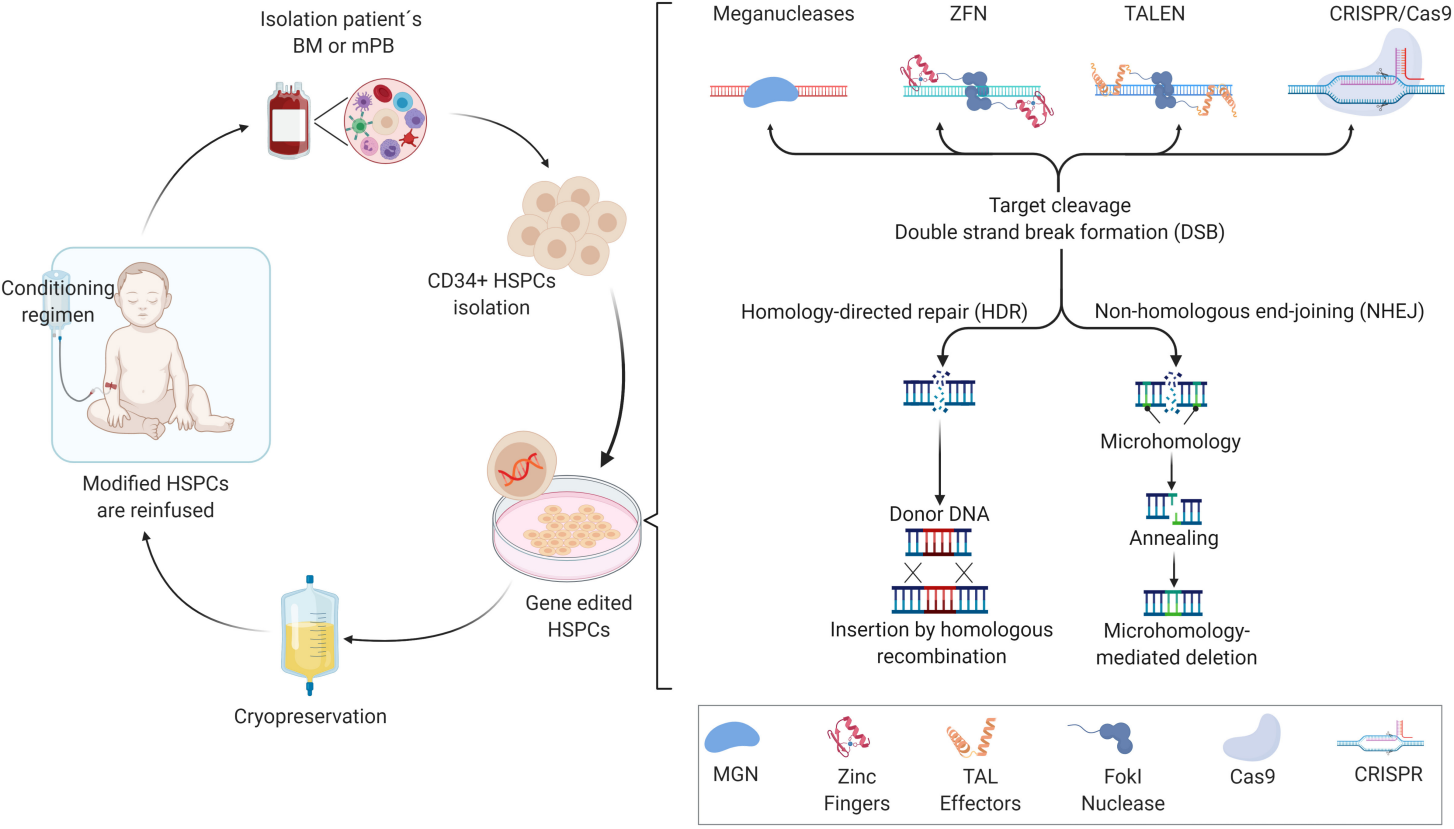
Representation of *ex vivo* HSCs gene editing, showing the crucial steps of the process. After harvesting the hematopoietic stem and progenitor cells (HSPCs) from mobilized peripheral blood or bone marrow, the CD34^+^ cells are enriched and cultured *ex vivo* in the presence of growth factors, which allows the maintenance and expansion of self-renewing stem cells, and are then subjected to gene editing tool transfer (e.g., meganucleases, ZFNs, TALENs, or CRISPR/Cas-derived nucleases). When the nuclease induces a standard DNA double-strand break (DSB) at the desired genomic *loci*, the homology-directed repair (HDR) machinery are recruited in order to repair the DNA, where a template donor DNA is supplied for the homologous recombination between the template and chromosomal DNA, or by non-homologous end-joining (NHEJ) without a homologous template DNA, resulting in small indels generation (insertions and deletions) if there is only one cut, or triggers large DNA deletions if two cuts. After the treatment, the patient receives a specific conditioning regimen that depletes endogenous HSPCs from the bone marrow and makes space for the *ex vivo* engineered cells to engraft. The gene-corrected cells are then reinfused intravenously and engraft in the bone marrow.

Allogeneic-hematopoietic stem cell transplants (allo-HSCT) have been used since the late 1960's to offer a potential lifetime cure for a variety of monogenic hematological diseases (Thomas et al., 1975). The main benefit of successful allo-HSCT is that the patient is cured for life, highlighting the concept that transplantation of healthy donor-derived HSCs containing the correct gene variant can reconstitute a functional hematopoietic system. While allo-HSCT can cure multiple blood and immune system disorders, clinical problems remain due to the challenge of finding a suitable HLA-matched bone marrow donor together with need for strong conditioning regimens for HSC engraftment, potentially resulting in subsequent complications such as graft-vs.-host disease (GvHD) or incomplete reconstitution of blood cell lineages. Moreover, chemotherapeutic conditioning regimens may result in infertility or development of lymphomas later in life. In patient genotype-specific cases when a suitable HLA-matched donor is not available, mismatched related donors are often used, however at the cost of increased morbidity and incomplete immune recovery leading to lower quality of life. To overcome these limitations of allo-HSCT, researchers initially have developed retroviral vectors that carry a recombinant version of the correct gene for permanent transfer into autologous CD34^+^ cell-enriched HSCs that. The *ex vivo*, genetically modified CD34^+^ cells that include HSCs are infused back into the patient and the genetically modified cells engraft and subsequently produce hematopoietic cells expressing the therapeutic gene (Figure 1). This *ex vivo* gene therapy principle has been shown to be efficacious in diseases such as severe combined immunodeficiency due to adenosine deaminase deficiency (ADA-SCID) (Aiuti et al., 2009), X-linked severe combined immunodeficiency (Hacein-Bey-Abina et al., 2002, 2010; Pavel-Dinu et al., 2019) and more recently for hemoglobinopathies including SCD, conditions that require high levels of therapeutic gene expression to attain phenotypic rescue (Woods et al., 2006; Badat and Davies, 2017). Currently, departing from “classic” gene therapy, gene editing technology based on programmable nucleases is offering the perspective for changing the genome of HSCs with unprecedented specificity and accuracy. Together with increased knowledge of the mechanisms that regulate human hematopoiesis, this has created the possibility to further developing cell and gene therapies for inherited diseases of the blood cell compartment. Backed by many years of fundamental research and, at times serendipity, the discovery of restriction enzymes was followed by that of other classes of DNA-modifying tools, including site-specific recombinases and programmable nucleases, such as meganucleases (MGN), zinc-finger nucleases (ZNFs), transcription activator-like effector (TALE) nucleases (TALENs), and more recently, powerful RNA-guided nucleases based on clustered regularly interspaced short palindromic repeats (CRISPR)-CRISPR-associated endonuclease (Cas) systems (Chandrasegaran and Carroll, 2016; Chen and Goncalves, 2018). In this context, the non-integrating adeno-associated vector (AVV) has become a widely exploited vehicle of donor DNA template that is required for homology directed repair (HDR) in HSCs (Bak et al., 2018). Single-strand and double-strand oligodeoxynucleotides (ODN) are also emerging as effective means to deliver donor template for HDR in many clinical relevant settings (Chen et al., 2015). The engineering of meganucleases with new DNA-binding specificities has been challenging in large part due to the fact that the DNA recognition and cleavage sites are located in the same domain. In contrast to the meganucleases, the DNA binding domains of ZFNs and TALENs are distinct from that of their FokI cleavage domains whose (catalytic) activation depends on target DNA binding of a working ZFN or TALEN pair resulting in local dimerization (Urnov et al., 2010). The ZFNs and TALENs DNA-binding domain consist of zing-finger motif and TALE repeat arrays, respectively, with each zinc-finger motif binding to specific nucleotide triplets and each TALE repeat recognizing individual single nucleotides. The changes of the zinc-finger motifs can be done by the nucleotides that are surrounding its triple target. As a consequence of this sequence context dependency, generating robust and highly specific ZFNs often requires complex protein engineering methods involving reiterative optimization cycles and/or screening of large zinc-finger libraries (Cathomen and Keith Joung, 2008). The straightforward TALE repeat-to-nucleotide one-to-one recognition code together with the fact that binding of a TALE repeat to its target nucleotide is not substantially altered by neighboring nucleotides (Mussolino and Cathomen, 2012), makes the assembly of functional and highly specific TALENs easier and more flexible than that of ZFNs (Jinek et al., 2013). While each programmable nuclease platform is at different stages of clinical development, RNA-guided CRISPR/Cas-based systems are becoming the tools of choice for pursuing genetic therapies based on genome editing principles and technologies. This principally stems from their high efficiency and increasingly improving specificity, as well as from their versatile RNA-dependent programmability and easy-to-use versatile design.

CRISPR sequences together with CRISPR-associated (Cas) protein genes form CRISPR/Cas loci as part of the adaptive immune systems in prokaryotes organisms, evolved as a strategy to fend off infectious agents, e.g., bacteriophages and foreign plasmids (Horvath and Barrangou, 2010; Wiedenheft et al., 2012; Rath et al., 2015). Scientists have been investigating the properties of these exquisite defense mechanisms encoded in various CRISPR loci for over 20 years. Crucially, in 2012, the real potential of CRISPR/Cas systems for genomic engineering purposes was uncovered in seminal studies by Gasiunas et al. (2012) and Jinek et al. (2012). In particular, through these eminent *in vitro* biochemical studies, these teams found that Cas9 proteins from *Streptococcus thermophilus* and *Streptococcus pyogenes*, respectively, are RNA-programmable site-specific endonucleases. Later, the CRISPR system was readily adapted by independent research groups that had the aim of turning the technique into a powerful genome editing platform for genome editing purposes in mammalian cells (Cho et al., 2013; Cong et al., 2013; Jinek et al., 2013; Mali et al., 2013).

Key adaptations involved codon-optimization of Cas9 reading frames encoding nuclease localization motifs and fusion of native trans-activating CRISPR RNA and CRISPR RNA moieties to form a so-called single-guide gRNA (sgRNA). The latter component binds to the Cas9 protein and address it to a target sequence consisting of a protospacer adjacent motif (NGG; in the case of *S. pyogenes* Cas9) and a typically 20 nucleotide-long sequence complementary to the 5′ end of the sgRNA (spacer). Upon target site binding and sgRNA-DNA hybridization, the HNH and RuvC-like nuclease domains of Cas9 become active resulting in site-specific DNA cleavage, of inducing double-stranded DNA (dsDNA) breaks at a specific genomic target region, homologous to the crRNA spacer sequence.

Two major DNA repair pathways exist in humans. The endogenous non-homologous end-joining (NHEJ) and homology-directed repair (HDR) pathways are responsible for repair of the double-stranded chromosomal breaks made by programmable nucleases allowing for the removal or insertion of new genetic information at specific genomic loci (Jinek et al., 2012). Typically, NHEJ processes are exploited for knocking-out preexisting genetic information after the exclusive transfer of programmable nucleases, whilst the HDR mechanism is mostly used for knocking-in new genetic information after the delivery of programmable nucleases together with exogenous (donor) DNA templates.

The prokaryotic-CRISPR/Cas9 genome editing tool has changed our ability to change and manipulate specific sequences of DNA and RNA in living cells from diverse species, including mammalian cells. The CRISPR/Cas9 system for genetic engineering is an exciting advancement for HSC gene therapy, although it potentially comes with safety risks, such as suboptimal specificity correlated with off-target effects and on-target but unwanted mutations, immunogenicity, and unfavorable bio-distribution.

## The Challenges of Genome Engineering in HSCs

**Figure 2:**
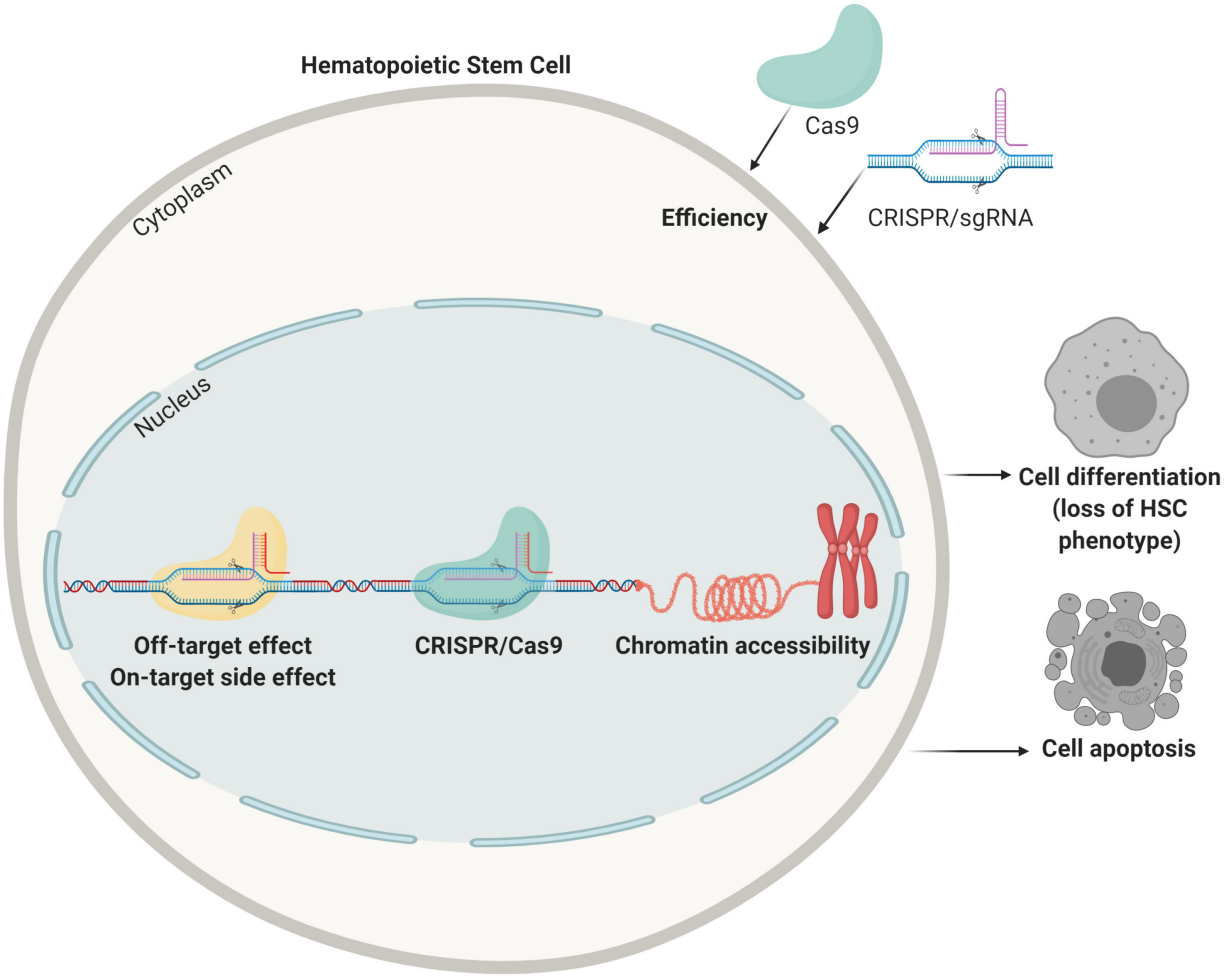
Points of improvement in CRISPR mediated gene correction in HSCs. Efficiency of delivery, off-target effects (targeting the wrong locus), side effects on the target sites (unwanted indels, translocations, and mutations), lack of accessible chromatin, apoptosis due to the harsh procedures, and loss of stemness are all problems that need to be tackled before obtaining clinically relevant HSC numbers that can be transplanted in patients.

Genome-editing tools in the form of the aforementioned programmable nucleases and their derivatives can, in principle, be projected for correcting or disrupting any disease-causing gene typically via knocking-in and knocking-out exogenous and endogenous DNA sequences, respectively, or via the introduction of specific point mutations (Byrne et al., 2014). HSCs are optimal target cells for therapeutic genome editing technologies owing to their self-renewal and differentiation capabilities (Hoke et al., 2012; Liu et al., 2012; Lee et al., 2019). However, these genome editing tools and strategies are initially mostly developed and tested in transformed cell lines that differ from their primary cell counterparts in key aspects, such as transfectability, cell death propensity, loss of differentiation capabilities, ploidy, and chromatin accessibility (Figure 2). Primary cells, unlike immortalized or full-fledged transformed cells, for the most part maintain their biological identity in proper culture systems, yet, they can only be propagated for a few generations *in vitro* before reaching senescence and, in the case of true HSCs, they are difficult to expand *in vitro*.

Hence, when thinking about applying these genome editing tools and strategies to primary cells, and in particular HSCs, one faces numerous challenges associated with the aforementioned intrinsic characteristics of these target cells and the sub-optimal performance of gene editing procedures, such as on-target and off-target side effects, as well as insertional mutagenesis risks and unregulated transgene expression resulting from random chromosomal integration of exogenous (donor) DNA templates (Crisostomo et al., 2006). In order to meet the safety requirements and other important criteria such as a high efficacy, high quality and good reproducibility, it is crucial that the genome-editing tool is proper developed and tested in appropriate cell types.

One of the most challenging issues of *ex vivo* genome-editing of HSCs, besides the low viability and the decreased differentiation potential of these cells upon prolonged culture, is the difficulty in achieving high gene delivery efficiencies. Part of the difficulty is the absence of methods that permit the *in vitro* identification and, thus, selection of *bona fide* HSCs (gene-modified or otherwise) from cultured hematopoietic cells. Another component of the difficulty concerns the gene transfer into HSCs. Because the existing protocols do not employ drug selection, the gene transduction methods need to yield enough functionally reconstituted cells for a good therapeutic response. Another limitation when applying genome-editing in HSCs is the low transplanted cell engraftment capacity due to their poor viability after gene-editing, especially when high percentages of non-edited cells are present after the *ex vivo* modification (Naldini, 2019). For those cases, the enrichment of the CD34^+^ fraction using a combination of additional hematopoietic surface markers may be important for the improvement of cell engraftment and repopulation, although these additional cell manipulations might lead to loss of stemness and cell death. Along these lines, it is necessary to identify which specific gene-editing tools and strategies are the most appropriate for each disease, and consider whether, on the basis of the disease phenotype, the modified cells present a selective advantage that might reduce conditioning regimens and increase the cell engraftment capacity.

## Genome Editing Techniques: Transition to the Clinic

The introduction of gene-editing tools in the form of engineered nucleases has provided strong support to the idea that targeted genetic therapies for treating monogenetic diseases of the hematopoietic system is achievable. Yet, there are multi-tier bottlenecks on the path to transitioning from applying HSC-directed gene-editing laboratory technologies to the clinic. To overcome these bottlenecks it will be crucial to develop and combine delivery agents and gene-editing reagents that allow for efficient and precise gene-editing at the HSCs level. Further, these integrated gene editing procedures need to be scalable under good manufacturing practice conditions, and, clearly, neither cytotoxic, or genotoxic. Moreover, there are other points that should have important improvements, such as the delivery of the homologous donor templates and the nuclease of choice.

Regardless of their class, programmable nucleases are capable of achieving high specificity, especially once individual reagents are identified and optimized for cleaving target sequences and not off-target sites (Akcakaya et al., 2018), but it is important to know that none of them are perfect. However, even when using the highly specific nucleases, when making changes at the desired target site, unintentional changes can be induced elsewhere in the genome due to, for instance, differences in nuclease amounts and chromosomal accessibility in different cell types. Indeed, these parameters might influence DNA cleavage and NHEJ-mediated repair (profiles) at secondary sites (White et al., 2017). These unwanted genome-modifying events present a modest hazard in experimental systems, where conclusions can be validated by (i) comparing independent gene-edited cells and organisms, (ii) “cleaning-up” the genetic background by out-breeding/cross-breeding and (ii) complementing gene knockouts via introducing wild-type gene sequences. However, for therapeutic applications off-target effects are more problematic. Methods have been developed for detecting, locating and quantifying those off-target effects (Koo et al., 2015). When applied in human therapy, we need to be assured that the adverse effects of the treatment are as minimal as possible while the one originally addressed gene is repaired.

Besides off-target effects, adverse effects caused by cleavage at the desired side of modification have also been reported (Kosicki et al., 2018; Chen et al., 2020). These unwarranted on-target effects can affect not only the genotype but also the phenotype of gene-edited cells (Chen et al., 2020) and are more difficult to assess, but clearly are undesired. In addition, as aforementioned, the efficiency of gene modification can be reduced due to the limited accessibly of target sequences tightly packed in heterochromatic regions (Chen et al., 2016; Daer et al., 2017), resulting in a lack of efficient delivery of the Cas enzyme or the DNA template needed for repair via homologous recombination. The limited access of gene editing reagents to the DNA can perhaps be overcome at some target loci by forcing the HSCs to enter into the S and M phases. However, *ex vivo* proliferation of HSCs without losing their stemness properties is still a daunting task (Tajer et al., 2019).

Despite these problems, researchers have reported significant advances in gene editing of HSCs for SCID. For instance, Genovese et al. have shown that gene editing for X-SCID is in principle possible (Genovese et al., 2014). In this report, ZFNs were used and the efficacy was relatively low, but some correction was obtained in human long-term repopulating HSCs transplanted in immune-deficient mice. The next improvement consisted of using RGN nucleofection for introducing an *IL2RG* transgene delivered via an adeno-associated viral vector pseudo-type (i.e., AAV6) into the first exon of the *IL2RG* gene that is deficient in X-SCID (Pavel-Dinu et al., 2019). The reported gene correction efficiencies were much higher but the phenotypic differences between corrected and uncorrected HSCs transplanted were only minor, with modest increases in T and NK cells, the two lineages affected in this type of SCID (Pavel-Dinu et al., 2019). This indicated that even for a relatively easy target such as an X-linked gene which only requires correction in one allele, efficacies need to be significantly improved for clinical application. Gene editing is particularly attractive for diseases where the expression of affected gene normally is strictly regulated. While gene addition approaches work well for X-linked SCID (Hacein-Bey-Abina et al., 2002; Pavel-Dinu et al., 2019), ADA-SCID (Aiuti et al., 2009), Wiskott–Aldrich syndrome (WAS) (Braun et al., 2014), RAG1-SCID (Garcia-Perez et al., 2020), and b-globin disease (Dong and Rivella, 2017), for IL7Rα-SCID and for the Hyper IgM syndrome (caused by mutations in the *CD40L* gene), gene addition with constitutively expressing vectors will cause severe side effects (Kuo et al., 2018). However, also for diseases caused by defects in such genes significant progress is being made. Indeed, Kohn et al. reported specific insertion of a recombinant *CD40L* sequence downstream of the endogenous *CD40L* promoter using RGNs and an AAV-delivered donor template (Kuo et al., 2018). Relevant levels of gene modification were achieved in primary HSCs and in patient-derived T cells. Therefore, significant progress is made to clinical implementation of these techniques. Nevertheless, clinical trials using CRISPR and HSCs have been confined to gene deletion strategies rather than editing of mutant genes. Examples include a gene disruption approach to delete the CCR5 HIV coreceptor and the disruption of erythroid lineage-specific enhancer of the BCL11A suppressor protein in the g-globin gene to induce re-expression of fetal g-globin in thalassemia patients (NCT03745287) (Psatha et al., 2018). Indeed, for bona fide gene editing in Hyper IgM syndrome due to CD40L mutations, T cells rather than HSCs are being proposed as target cells in clinical trials.

## Regulatory Authorities

In the last three decades, the *ex vivo* gene therapy in HSCs has been progressing substantially from the pre-clinical stage to clinical trials (Thrasher and Williams, 2017; Staal et al., 2019). With the FDA-approved first clinical trial gene-editing of HSCs for the treatment of HIV using the ZFNs CCR5 (Tebas et al., 2014), a new paradigm treatment in cell and gene therapy had been started. Before wide-spread clinical approval, however, there are several regulatory hurdles. Regulation may be complex and vary across countries and continents because gene-editing medicine entails the unprecedented introduction of designed alterations in the genetic make-up of some of the patient's cells. As a minimum, regulators will focus on whether the gene disruption/restoration is based on robust preclinical evidence, as illustrated by the US FDA approval of multiple clinical trials.

Although there is a great promise for gene-editing in the future of medicine, the regulatory approval by the competent authorities will not be granted in the short term. One of the reasons is because the authorities strictly guard safety and well-being of patients (White et al., 2017). One of the major obstacles is that there is no clear consensus regarding the occurrence of on-target and off-target alterations by the gene-editing tools, and also it is not clear when and how these effects should be monitored in the clinical applications (Joung, 2015). Regulatory authorities and the pharmaceutical industry of Europe, Japan, and the USA have developed some consideration documents regarding gene therapy (Coppens et al., 2018; de Wilde et al., 2018), indicating that more regulatory harmonization is indispensable in order to realize the therapeutic benefits of genome editing worldwide.

The versatility and robustness of gene-editing approaches are expected to positively contribute to the development of novel somatic disease treatments. However, the technology could also lead to some unfavorable social phenomena due to high prices and public misconceptions. The general public should understand that so far only a few gene therapy products have been approved by health regulators worldwide. Moreover, scientists have the obligation to provide the public with accurate and realistic information regarding the prospects, as well as the problems associated with the use of somatic gene-editing therapy. In addition, good communication between researchers and the regulatory authorities are key to fulfill the promises and to achieve the medical benefits of genome editing. Communication and cooperation should foster an increase in worldwide regulatory harmonization. This should eventually lead to clinical benefit for those affected with inborn diseases.

## Final Remarks

Tremendous progress has been made in the field of gene editing over the last few years. However, no clinical trials using this technology have been used so far to treat immune deficiencies via gene editing for reasons of efficiency and safety. To have HSC gene editing working safely at the scale needed for clinical application remains challenging and will require carefully designed protocols using the correct target cells, assays to detect potential side effects, and comparisons with more conventional allo-HSCT and gene addition therapy methods. Such efforts will hopefully lead to the clinical application of gene editing techniques to cure monogenetic diseases of the hematopoietic system.

## Author Contributions

All authors listed have made a substantial, direct and intellectual contribution to the work, and approved it for publication.

## Conflict of Interest

The authors declare that the research was conducted in the absence of any commercial or financial relationships that could be construed as a potential conflict of interest.
